# Braithwaite’s Retrospect of Practical Medicine and Surgery

**Published:** 1848-10

**Authors:** 


*Braithwaite's Retrospect of Practical Medicine and Surgery.* Part the seventh, New York: Daniel Adee, 107 Fulton street.

*The half Yearly Abstract of the Medical Sciences, etc.* Edited by W. H. Ranking, M. D., etc. No. seven, Philadelphia: Lindsay & Blackiston.

These valuable semi-annuals continue to make their appearance in due season, being promptly republished in this Country, and are, we believe, as acceptable as ever, to the profession.

